# Omega-3 fatty acids prevent gestational diabetes mellitus via modulation of lipid metabolism

**DOI:** 10.1515/biol-2022-0928

**Published:** 2024-08-06

**Authors:** Xuan Zhang, Fang Li, Botao Yang, Wei Zhang, Yingchun Wang

**Affiliations:** Department of Obstetrics, Shijiazhuang Obstetrics and Gynecology Hospital, Shijiazhuang, Hebei Province, 050011, China; Department of Gynecology and Obstetrics, Langfang Health Vocational College, South of Siguang Road, Dongfang University Town, Langfang Economic and Technological Development Zone, Langfang, Hebei Province, 065001, China

**Keywords:** gestational diabetes mellitus, omega-3 fatty acids, lipid metabolism, glucose metabolism

## Abstract

The incidence rate of gestational diabetes mellitus (GDM) remains high among pregnant women in the second trimester of pregnancy. However, the main clinical approach to alleviate the symptoms of GDM is to control the diet. Our study explored the therapeutic effects of omega-3 fatty acids (ω-3 FAs) on GDM at the cellular and animal levels. We found that ω-3 FAs can promote the transformation of M0 macrophages into anti-inflammatory M2 macrophages. The transformed M2 macrophages promoted β-oxidation and reduced hepatocyte lipid synthesis (*P* < 0.05), thereby promoting hepatic function and preventing the excessive accumulation of lipid droplets in the hepatocyte cell line HepG2. Supplementation of ω-3 FAs in pregnant GDM mice significantly reduced fasting blood glucose levels, glucose tolerance test, and insulin tolerance test indices, and lipid accumulation in the liver and effectively prevented the occurrence of liver fibrosis (*P* < 0.05). These therapeutic effects may be mediated through the anti-inflammatory effects of ω-3 FAs (*P* < 0.05). ω-3 FAs also had positive effects on the offspring of pregnant GDM mice, as demonstrated by reduced birth mortality and improved glycemic stabilization (*P* < 0.05). In conclusion, this study provides a possible translational medicine strategy for the treatment of GDM.

## Graphical abstract

**Figure:**
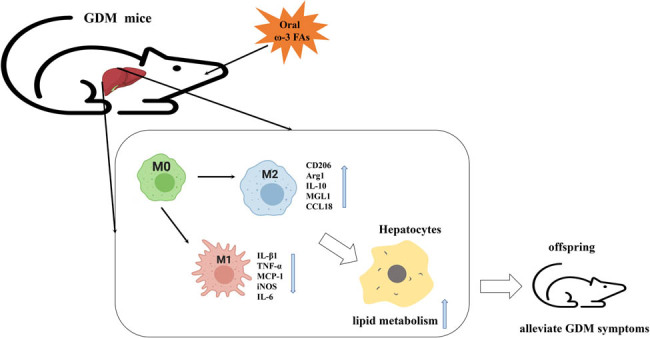

## 1 Introduction

Gestational diabetes mellitus (GDM) has a high incidence during pregnancy, seriously affects the life safety and health of pregnant women, and may affect the fetus. The global prevalence of GDM is as high as 2–25% globally, and this wide range of incidence may be due to inconsistent evaluation criteria worldwide [1,2,3]. GDM mainly occurs during the second or third trimester [4,5]. Pregnant women with GDM exhibit glucose intolerance and insulin, vitamin D, and omega-3 fatty deficiencies [6,7]. Insulin secreted by the pancreas of pregnant women with GDM is insufficient to resist the insulin inhibition effect caused by hormones produced by the placenta, such as estrogen, human placental lactogen, and cortisol [8]. The condition persists from the time of the first diagnosis with GDM until childbirth [9]. Many factors are associated with GDM, such as obesity, non-white ethnicity, increased maternal age, family history of diabetes, and history of giving birth to large infants. GDM even has long-term effects on pregnant women after childbirth, with an incidence of developing type 2 diabetes as high as 50% after 5 years of pregnancy and even 70% after 22–28 years of pregnancy [10]. Additionally, pregnant women with GDM consistently provide a high-glycemic environment for the fetus, which may increase the probability of neonatal obesity, metabolic syndrome, and other cardiometabolic disorders in the offspring [11]. Therefore, it is critical to determine a suitable treatment strategy for GDM.

Omega-3 fatty acids (ω-3 FAs) are essential for human health, and their deficiency can cause several chronic diseases [12]. ω-3 FAs, including eicosapentaenoic acid (EPA), docosahexaenoic acid (DHA), and other family members, are reported to have anti-inflammatory effects and can produce therapeutic effects on inflammatory diseases, including diabetes [13,14]. ω-3 FAs can inhibit the inflammatory effects of macrophages by binding to the G protein-coupled receptor 120 (GPR120) [15]. Inflammation is the primary cause of insulin resistance in patients with diabetes. Feeding ω-3 FAs to diabetic mice can effectively inhibit inflammation and enhance systemic insulin sensitivity [16]. Additionally, GPR120 is also highly expressed in hepatocytes, and supplementation mice with ω-3 FAs improves the accumulation of lipid in the liver caused by a high-fat diet (HFD) [17,18]. Then, the anti-inflammatory effect of ω-3 FAs is finally demonstrated in the inhibition of pro-inflammatory factor IL-1β production [19].

Since GDM is often accompanied by blood ω-3 FAs deficiency, and ω-3 FAs have a role in regulating glucose and lipid metabolism, we speculated that ω-3 FAs may alleviate the symptoms of GDM in pregnant women. HFD combined a low dose of streptozotocin (STZ) is an ideal method for establishing a type 2 diabetes model [20]. Elevated levels of blood glucose, blood insulin, blood triglyceride, and cholesterol (CHO) in mice are consistent with the pathogenesis and clinical characteristics of type 2 diabetes in humans [21]. Thus, the HFD combined with a low dose of STZ was used to induce a GDM mouse model. Inflammation plays an important role in abnormal glucose and lipid metabolism [22]. Among all immune cells, macrophages can effectively regulate the lipid metabolism, and ω-3 FAs can influence macrophage transformation [23,24]. In this study, macrophages induced by ω-3 FAs were co-cultured with hepatocyte cell line HepG2 to observe the effect of inflammation on glycolipid metabolism. The completion of this study may provide novel insights and basic evidence for the therapeutic intervention of GDM.

## 2 Materials and methods

### 2.1 THP1 cell culture

The human leukemia monocytic cell line THP1 was maintained at 2–3 × 10^5^ cells/mL in RPMI 1640 medium supplemented with 10% FBS and 2 mmol/L l-glutamine. M0 phenotype macrophages were induced by the addition of 200 nM phorbol 12-myristate 13-acetate (PMA, Sigma-Aldrich, USA) for 3 days [25]. The PMA-treated cells were further stimulated to differentiate using 10 ng/mL ultra-pure LPS (Sigma-Aldrich) for 8 h [26]. After the induction, 20 μM DHA was added into the culture for another 20 h [27]. Then, the cells or supernatants were collected for further experiments. For the co-culture system, the macrophages were moved to the HepG2 culture well, and the ω-3 FAs were additionally added.

### 2.2 HepG2 cell culture

HepG2 cells were seeded and cultured in media consisting of EMEM (ATCC 30-2003), 10% FBS (Gibco), and 1% penicillin and streptomycin (Gibco) until specific treatments were applied. Palmitic acid (PA, Sigma-Aldrich) and oleic acid (OA, Sigma-Aldrich) were resolved in ethanol to a final concentration of 0.5 mM and then sonicated (200 W, 4 s on bursts and 6 s interval) on ice, and then the emulsion was stocked. Before use, the emulsion stock solutions were dissolved in a complete medium at 60°C to a concentration of 0.5 mM. After cooling to 37°C, the medium was used to induce fatty acid production in the HepG2 cells.

### 2.3 Cell proliferation assay

The proliferation of HepG2 cells was measured using a CCK-8 kit (Beyotime). Cells (5 × 10^3^ cells/well) were seeded into 96-well plates and co-cultured with or without ω-3 FAs for 0, 24, and 48 h. Then, 10 μL of CCK-8 solution was added to the plates. After incubation for 4 h at 37°C, the absorbance at 450 nm was recorded on a microplate reader (Thermo Fisher Scientific).

### 2.4 GDM model establishment

C57BL/6 J mice (8 weeks old) were fed at 25℃ with 12 h light–dark cycles. Female mice were fed a HFD; 60% kcal) for 4 weeks before mating and maintained on it until delivery. Two female mice were mated overnight with one male mouse. The presence of vaginal plugs was treated at the start time of pregnancy and defined as gestational day 0. For the GDM model group (*n* = 12) [28], 30 mg/kg STZ, dissolved in citrate buffer, was injected intraperitoneally into the mice each day from gestational days 1 to 4. Control mice (*n* = 6) were normal pregnant mice fed a normal chow diet throughout the whole process and were injected with an equal volume of citrate buffer from gestational days 1 to 4. Then, 3 days after the last time STZ or citrate buffer injection, random blood glucose level was tested from the tail vein using a glucometer (Roche, USA). Then, mice with GDM were randomly divided into two groups: the GDM group (*n* = 6) and the GDM with the ω-3 FAs treatment (ωGDM) group (*n* = 6). The ωGDM group was given DHA 50 mg and EPA 100 mg daily by oral administration [29]. An equal volume of normal saline was intragastrically administered to the GDM group. On gestational day 18, some mice were sacrificed to collect the blood and livers. The other mice were allowed to give birth naturally to estimate offspring outcomes. All the animal experiments conformed to the guidelines for the Care and Use of Animals published by the Institutional Animal Ethics Committee.


**Ethical approval:** The research related to animal use has been complied with all the relevant national regulations and institutional policies for the care and use of animals and has been approved by the Animal Ethics Committee of Shijiazhuang Obstetrics and Gynecology Hospital.

### 2.5 Glucose tolerance test (GTT) and insulin tolerance test (ITT)

Mice were fasted for 16 h, followed by intraperitoneal injection of 1.5 g/kg body weight glucose dissolved in saline for the GTT. Tail vein blood glucose levels were measured using a glucometer at 0, 15, 30, 60, 90, and 120 min after injection. Mice were first fasted for 6 h, followed by an intraperitoneal injection of 0.5 U/kg body weight insulin for the ITT. Tail vein blood glucose levels were measured using a glucometer at 0, 15, 30, 60, 90, and 120 min after injection.

### 2.6 Measurement of insulin, adiponectin, and inflammatory factor levels

After fasting for 16 h, 100–200 μL tail vein blood was collected at gestational day 18. Blood samples were centrifuged at 2,000 rpm for 20 min to separate the serum and then stored at −80°C. The insulin, adiponectin, TNF-α, MCP-1, IL-1β, and IL-8 levels in serum were tested by ELISA kits (CUSABIO, China) according to the manufacturer’s guidance.

### 2.7 Quantitative real-time PCR (qRT-PCR)

To extract total RNA, the cells and mouse liver tissues were incubated with TRIzol reagent (Solarbio, Beijing, China). RNA was reverse-transcribed to cDNA using a cDNA Synthesis Kit (Thermo Fisher Scientific, USA). SYBR Green PCR Master Mix (Thermo Fisher Scientific) was used for qRT-PCR detection using an Applied Biosystems 7300 instrument (ABI 7300, USA). GAPDH was used as the internal control. Relative gene expression levels were normalized to the 2^−ΔΔCt^. The primer sequences used are listed in Table S1.

### 2.8 Hematoxylin-eosin (HE), oil red, and sirus red staining of mice liver

On gestational day 18, after collecting blood samples, the mice were euthanized, and their livers were collected for paraffin or frozen sectioning. Subsequently, the liver paraffin section (4 μm) was stained with HE for pathological changes under an optical microscope. For oil red staining, the frozen liver sections (8 μm) were subjected to oil red O according to the previous report [29], and the intracellular lipid droplets were observed and evaluated on a bright-field microscopy (Leica). Sirius Red staining was utilized to enhance the visualization of collagen fibers. Deparaffinized sections were subjected to an incubation process in Picro-Sirus Red solution (Abcam) for a duration of 1 h. Subsequently, the sections were rinsed twice with an acetic acid solution and dehydrated in absolute alcohol. The final step involved mounting the samples for microscopic examination. Images were then obtained using a CKX41 light microscope (Olympus).

### 2.9 Statistical analysis

All data are presented as the mean ± standard error of the mean. Differences between the two groups were analyzed using a two-tailed Student’s *t*-test. Differences among groups three or more groups were analyzed using one-way ANOVAs for multiple comparisons. Data analysis was performed using the GraphPad Prism software. *P*-values <0.05 were considered statistically significant.

## 3 Results

### 3.1 ω-3 FAs promote the transformation of M0 macrophage into anti-inflammatory M2 phenotype

**Figure 1:**
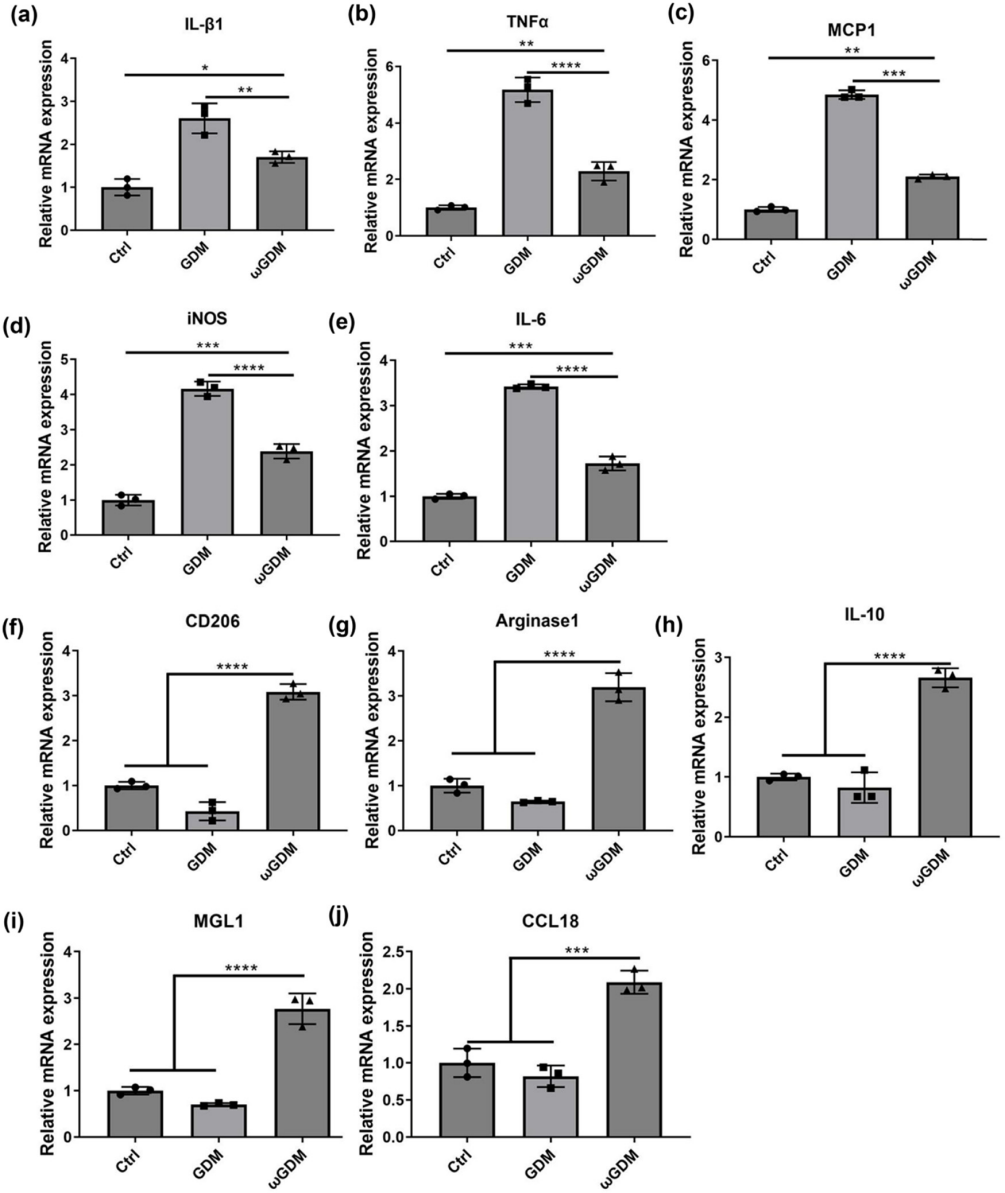
Effect of ω-3 FAs on macrophage transformation. (a)–(e) qRT-PCR detection of M1 macrophage markers (IL-β1, TNF-α, MCP-1, iNOS and IL-6). (f)–(j) qRT-PCR detection of M2 macrophage markers (CD206, Arginase1, IL-10, MGL1, and CCL18). *P < 0.05, **P < 0.01, ***P < 0.001, ****P < 0.0001 vs Ctrl or GDM, *n* = 3. Ctrl: control; GDM: gestational diabetes mellitus; ωGDM: GDM with the ω-3 FAs treatment.

We verified the effect of ω-3 FAs on macrophages at the cellular level. THP1 cells were used as model cells. After the induction of THP1 cells into M0 type, the medium containing LPS and IFN-γ was used for a short-term induction and then was replaced with the medium containing ω-3 FAs to further induce cell transformation. Experimental results showed that ω-3 FAs could effectively reduce the transformation of M0 cells into pro-inflammatory M1 macrophages (down-regulated expression of M1 macrophage markers IL-β1, TNF-α, MCP-1, iNOS, and IL-6 genes, as shown in Figure 1a–e) and promote transformation of M0 macrophages into anti-inflammatory M2 macrophages (M2 macrophage markers CD206, Arginase1, IL-10, MGL1, and CCL18 gene expressions were up-regulated, as shown in Figure 1f–j). These results indicate that ω-3 FAs can effectively inhibit inflammation.

### 3.2 Macrophages induced by ω-3 FAs promote lipid metabolism in hepatocytes

**Figure 2:**
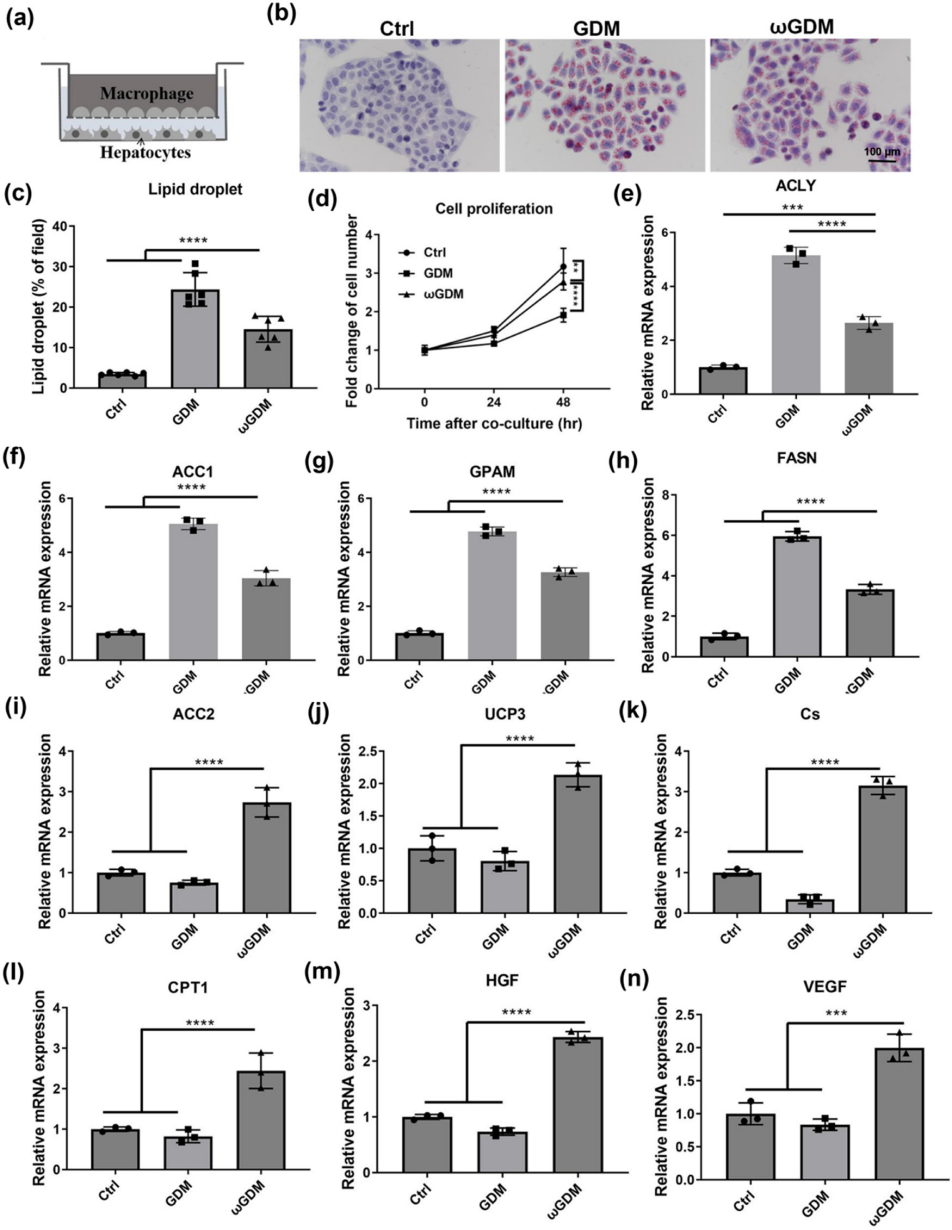
ω-3 FAs induced macrophages influence on lipid metabolism in hepatocytes. (a) Co-culture diagram of macrophages and HepG2 cells. (b) Oil red staining of HepG2 cells treated with OA and PA. (c) The proportion of lipid droplets. (d) Proliferation of HepG2 cells. (e)–(h) Expressions of genes related to fatty acid synthesis. (i–l) Expressions of β-oxidation related genes. (m and n) Expressions of growth factors, HGF and VEGF. **P < 0.01, ***P < 0.001, ****P < 0.0001, vs Ctrl or GDM. *n* = 3. Ctrl: control; GDM: gestational diabetes mellitus; ωGDM: GDM with the ω-3 FAs treatment.

Then, macrophages induced by ω-3 FAs were co-cultured with hepatocytes HepG2 (Figure 2a) to observe the effects of inflammation on glycolipid metabolism. The HepG2 cells were treated with OA or PA to simulate the metabolic status of the liver in women with GDM. The experimental results showed that OA and PA could make a large amount of lipid droplets accumulate in HepG2 cells, but after co-culture with macrophages induced by ω-3 FAs, lipid droplets accumulation was effectively alleviated (Figure 2b and c). The proliferative capacity of HepG2 is also affected by ω-3 FAs, demonstrating promoted cell proliferation (Figure 2d). We then assessed the lipid metabolism capacity of cells at mRNA levels and found that the expressions of lipid synthesis-related genes (ACLY, ACC1, GPAM, and FASN) were down-regulated in HepG2 after co-culturing them with macrophages induced by ω-3 FAs (Figure 2e–h). The expression of β-oxidation-related genes (ACC2, UCP3, Cs, and CPT1) were up-regulated (Figure 2i–l). Subsequently, the mRNA levels of HGF and VEGF, which characterize the hepatic function, were detected, and it was found that the expression levels of both genes were significantly increased (Figure 2m and n), indicating that ω-3 FAs could protect the function of hepatocytes.

### 3.3 ω-3 FAs relieve GDM symptoms

**Figure 3:**
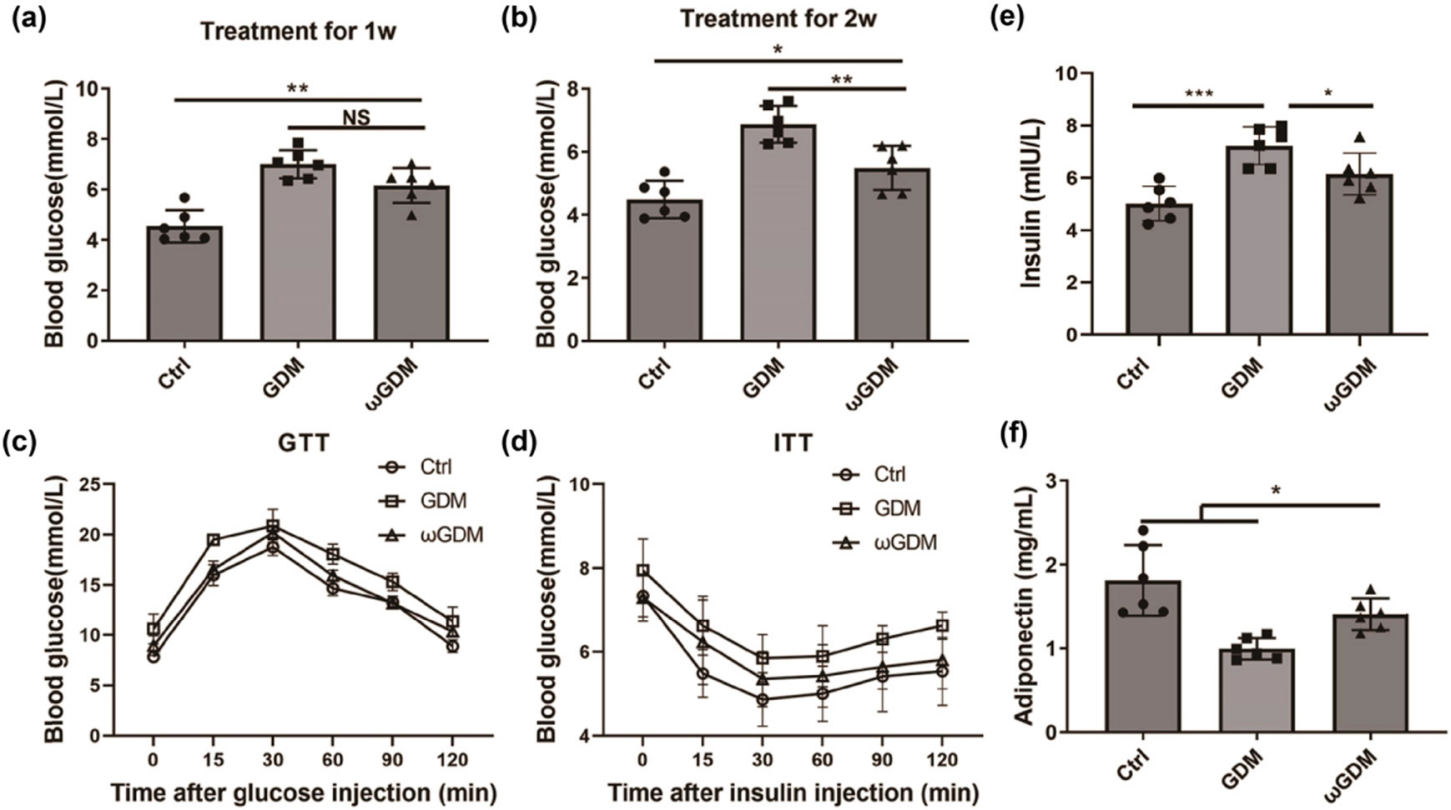
Effects of supplementation with ω-3 FAs on glucose metabolism in GDM pregnant mice. (a) Fasting blood glucose levels after 1 week of the ω-3 FAs treatment. (b) Fasting blood glucose levels after 2 weeks of the ω-3 FAs treatment. (c) After 2 weeks of treatment with the ω-3 FAs, GTT was detected in pregnant mice. (d) After 2 weeks of treatment with the ω-3 FAs, ITT detection was performed on pregnant mice. (e) Insulin levels in the serum of pregnant mice after 2 weeks of treatment with ω-3 FAs. (f) Adiponectin levels in serum of pregnant mice after 2 weeks of treatment with ω-3 FAs. *P < 0.05, **P < 0.01, ***P < 0.001, vs Ctrl or GDM. *n* = 6. Ctrl: control; GDM: gestational diabetes mellitus; ωGDM: GDM with the ω-3 FAs treatment.

After a series of *in vitro* cell experiments to confirm the function of ω-3 FAs, pregnant mice with GDM were given ω-3 FAs as supplement. After 1 week of treatment, although blood glucose concentration showed a downward trend, it was not statistically significant (Figure 3a). After 2 weeks of treatment, the blood glucose level was significantly downregulated (Figure 3b), indicating that continuous supplementation with ω-3 FAs could effectively normalize the blood glucose level of pregnant mice with GDM. GTT and ITT tests were performed on pregnant mice after 2 weeks of treatment, and it was found that blood glucose levels after treatment with ω-3 FAs were lower than those in pregnant mice with GDM without such a treatment (Figure 3c and d). Next, we detected the insulin level in the serum of pregnant mice and found that ω-3 FAs supplementation decreased the serum insulin level, indicating that ω-3 FAs may stabilize the blood glucose level by regulating insulin production (Figure 3e). Adiponectin level in the blood was also measured, and the results were similar to those for insulin. ω-3 FAs can increase the levels of adiponectin in blood (Figure 3f), indicating that ω-3 FAs also affect the lipid metabolism in pregnant GDM mice.

### 3.4 Effects of ω-3 FAs on liver function in GDM pregnant mice

**Figure 4:**
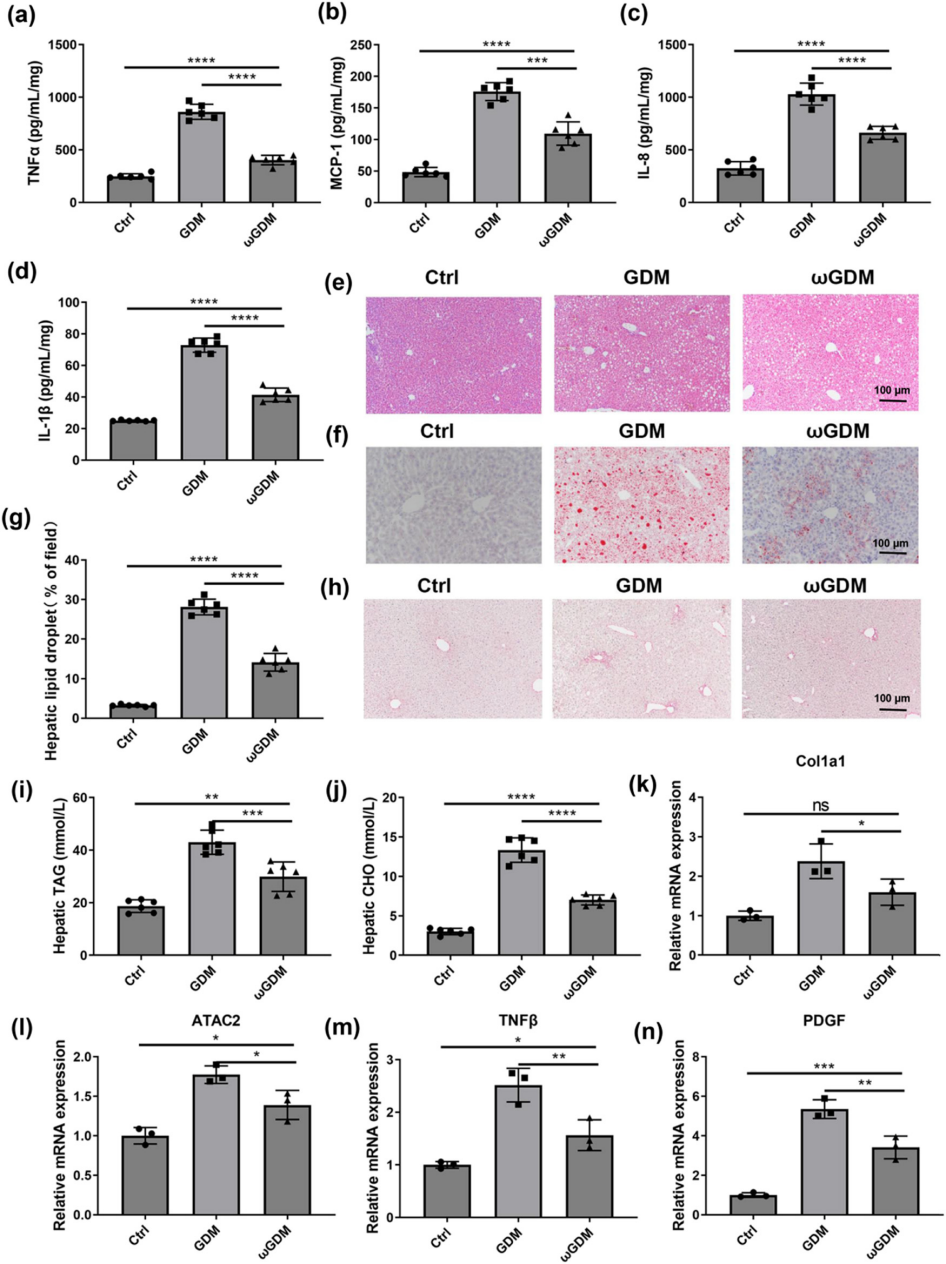
Effects of the ω-3 FAs treatment on lipid metabolism and inflammation in pregnant mice. Serum levels of inflammatory cytokines TNF-α (a), MCP-1 (b), IL-8 (c), and IL-1β (d). (e) HE staining of liver sections in pregnant mice. (f) Oil red staining of liver sections in pregnant mice. (g) Proportion of liver lipid droplets area in pregnant mice. (h) Sirius Red staining of liver sections of pregnant mice. (i) Liver TAG content in pregnant mice. (j) Liver CHO content of pregnant mice. (k)–(n) Expression levels of factors associated with liver fibrosis. *P < 0.05, **P < 0.01, ***P < 0.001, ****P < 0.0001, vs Ctrl or GDM. *n* = 6. Ctrl: control; GDM: gestational diabetes mellitus; ωGDM: GDM with the ω-3 FAs treatment.

In the following, we tested the serum levels of inflammatory factors in GDM pregnant mice after 2 weeks of treatment with ω-3 FAs. The results showed that the levels of TNF-α, MCP-1, IL-8, and IL-1β, the proteins with proinflammatory effects, were significantly decreased compared with those in GDM pregnant mice, indicating that the ω-3 FAs treatment reduces the systematic inflammatory response of GDM pregnant mice (Figure 4a–d). After 2 weeks of treatment, livers of the pregnant mice were collected for further examination. HE staining showed that the treatment of ω-3 FAs could effectively alleviate liver damage (Figure 4e). Then, we performed oil red staining on the liver sections and found that the treatment of ω-3 FAs significantly reduced the accumulation of lipid droplets in the liver (Figure 4f and g). Moreover, we conducted a quantitative analysis of the triglycerides (TAG) and CHO in the liver and found that both were significantly decreased (Figure 4i and j). These results suggest that supplementation of ω-3 FAs can regulate liver lipid metabolism. Then, Sirius red staining was performed on the liver, and there was no significant amount of collagen deposition in the livers of each group at the protein level (Figure 4h). Compared with the GDM group, mRNA levels of COL1 A1, ATAC2, TNF-β, and PDGF in the ω-3 FAs treatment group showed a significant decrease (Figure 4k–n), suggesting that the treatment with ω-3 FAs may effectively inhibit the occurrence of liver fibrosis.

### 3.5 Effects of supplementation with ω-3 FAs on offspring

**Figure 5:**
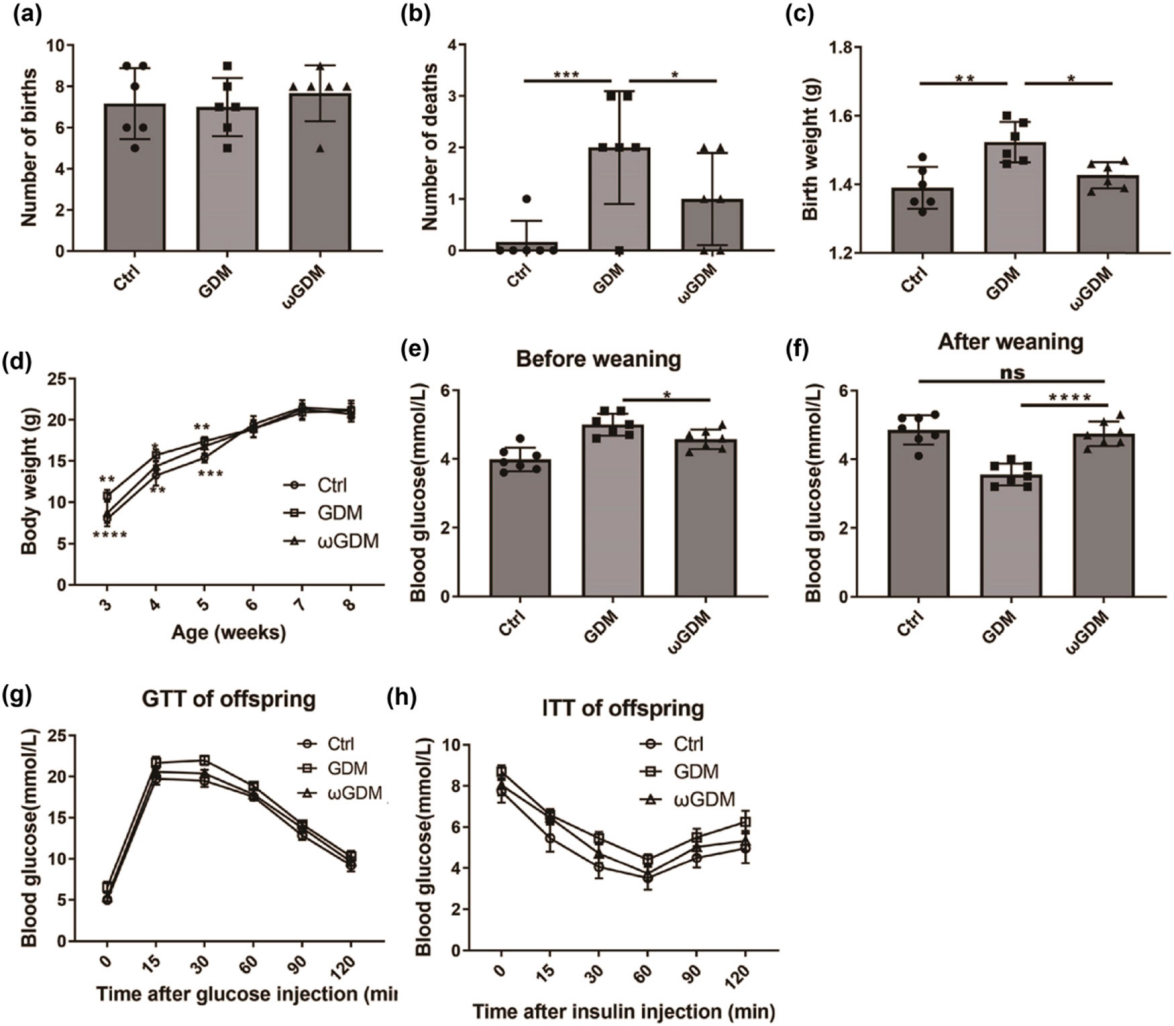
Effects of the ω-3 FAs treatment on offspring. (a) Number of newborn mice was recorded. (b) Number of neonatal mouse deaths. (c) Body weight of offspring. (d) Weight change in neonatal mice over time. (e) Fasting blood glucose levels in offspring before weaning. (f) Fasting blood glucose levels in offspring after weaning. (g) GTT detection of offspring at 8 weeks. (h) ITT detection in offspring at 8 weeks. *P < 0.05, **P < 0.01, ***P < 0.001, vs Ctrl or GDM. *n* = 6. Ctrl: control; GDM: gestational diabetes mellitus; ωGDM: GDM with the ω-3 FAs treatment.

We monitored the offspring in each group and found no statistically significant difference in the number of newborns (Figure 5a); however, supplementation with ω-3 FAs effectively reduced neonatal mortality (Figure 5b). The weight of newborn mice was measured, and it was found that ω-3 FAs treatment could reduce the giant newborn mice caused by GDM (Figure 5c). In order to explore whether the treatment with ω-3 FAs can have lasting effects on offspring, we carried out 8-week weight measurements and found that the weight of the offspring of pregnant mice treated with ω-3 FAs was lower than that of the offspring of GDM pregnant mice within 5 weeks after birth. However, from the sixth week, the weight of the offspring tended to be the same among the groups (Figure 5d), indicating that the influence of GDM on the offspring could persist for some time, but not for a lifetime. We then measured the blood glucose levels of the offspring before and after weaning. It was found that the blood glucose levels in the offspring of pregnant mice with GDM were higher than those of the offspring treated with ω-3 FAs, and the lowest after weaning (Figure 5e and f). It indicates that the breast milk affects the maintenance of blood glucose levels in the offspring. We tested the GTT and ITT in the 3-week-old offspring and found that the glucose metabolism level in the pregnant offspring treated with ω-3 FAs was more similar to that of the pregnant offspring in the control group (Figure 5g and h). These results indicate that the ω-3 FAs treatment can not only relieve the symptoms of GDM in pregnant mice, but also have a protective effect on the offspring.

## 4 Discussion

The incidence of GDM in pregnant women is high, and serious complications may occur. If not properly treated, the disease can have serious consequences for both the mother and fetus [30]. Existing data show that the occurrence of GDM is related to a variety of factors, such as vitamin D deficiency, ω-3 FAs deficiency, etc. [31,32]. However, there are few reports on whether the supplementation with ω-3 FAs can effectively relieve GDM symptoms in pregnant mice and reduce the adverse effects of hyperglycemic environment on the fetus.

The enrichment of ω-3 FAs can influence the transformation of macrophages and exert anti-inflammatory effects, as documented previously [19,24]. In this study, we found that macrophages treated with ω-3 FAs could effectively promote the transformation of M0 macrophages into anti-inflammatory M2 macrophages. Lipid droplet accumulation in HepG2 cells was reduced when co-cultured with M2 macrophages. Excessive accumulation of lipids can affect the function of hepatocytes, and an inappropriate intervention may develop into cirrhosis or even liver cancer [33,34]. This study discovered that the M2 macrophages induced by ω-3 FAs promoted the expression levels of HGF and VEGF, suggesting that ω-3 FAs can reduce lipid accumulation in liver and protect liver function by regulating the immune response. Similarly, a recent study clarified that ω-3 FAs affected the liver lipid metabolism and alleviated the progression of non-alcoholic fatty liver disease by decreasing the accumulation of CHO and triacylglycerols in a mouse model [18]. To date, whether ω-3 FAs affect lipid metabolism via regulation of macrophage polarization remains unclear. The present study is the first to demonstrate that the ω-3 FAs can reduce lipid accumulation in hepatocytes via promoting M2 macrophage transformation.

Omega FAs are potential biomarkers and are considered to be associated with a GDM risk [35]. Maternal fatty acid and lipid metabolism changes during pregnancy can promote fetal growth and development [36]. Then, we tested the therapeutic effects of ω-3 FAs in GDM pregnant mice and their offspring. We found that the ω-3 FAs treatment could relieve the symptoms of GDM and reduce the blood glucose level in pregnant mice under starvation conditions. Meanwhile, GTT, ITT, and serum insulin also showed a decreasing trend, and the adiponectin content increased. These results suggest that ω-3 FAs stabilize the blood glucose levels through the regulation of insulin. Mammals are unable to synthesize the precursors of ω-3 FAs, which are required for the proper function of human body cells and must be supplemented through food [37]. Our study demonstrated the therapeutic effects of ω-3 FAs in GDM pregnant mice and their offspring by stabilizing blood glucose levels via the regulation of insulin. Our data supports the study performed in humans, concluding that ω-3 FAs improve glycemic control and reduce the levels of triglycerides and inflammation [31,38,39]. The ω-3 FAs are known to inhibit inflammation and enhance systemic insulin sensitivity in diabetic mice. Based on this evidence, this study further found that ω-3 FAs exerted therapeutic effects on GDM pregnant mice and their offspring by regulating insulin to stabilize blood glucose levels, which has never been reported before.

Glucose metabolism is directly related to lipid metabolism, and an increase in adiponectin levels indicates that lipid metabolism is active [40]. We found that the treatment with ω-3 FAs could effectively reduce the lipid accumulation in the liver of GDM pregnant mice and inhibit the occurrence of liver fibrosis. Moreover, the treatment with ω-3 FAs could reduce the birth mortality in the offspring of GDM pregnant mice and weight of newborn mice. The effects of GDM on the offspring persist during lactation; however, they begin to disappear in the sixth week after birth, indicating that a high-glucose environment may affect the blood glucose homeostasis in the offspring. After the removal of the harmful environment, the offspring have the ability to spontaneously recover. Consistent with human studies, ω-3 FAs are widely used during pregnancy and GDM, and are beneficial in regulating maternal, fetal, and offspring metabolic function [31,41]. The pathogenesis of GDM mainly involves changes in lipid metabolism, glucose metabolism, and insulin resistance, and the offspring of GDM women are susceptible to high-glucose tolerance disorder. Insulin treatment is widely used for GDM, while the long-term usage of insulin might affect the developing fetus. In this case, ω-3 FAs supplementation may be a novel and effective strategy for the treatment of GDM.

In this study, we systematically analyzed the therapeutic effects of the ω-3 FAs on glucose and lipid metabolism in GDM pregnant mice and their offspring. We briefly explored the underlying mechanism, providing an alternative for translational medicine and theoretical support for the clinical application of the ω-3 FAs in the treatment of GDM. This study had some limitations. Exploration of the mechanism has mainly focused on the detection of the mRNA levels, and no confirmation on protein level was performed. The target for ω-3 FAs has not yet been explored. Although the effects of ω-3 FAs on macrophages have been verified *in vitro*, their levels in individual cells have not been explored *in vivo*. We will continue to improve on these aspects in future studies. This study provides evidence suggesting that ω-3 FAs can alleviate the symptoms of GDM.

## Supplementary Material

Supplementary Table
