# Supplementary material for: Omega-3 fatty acids prevent gestational diabetes mellitus via modulation of lipid metabolism
Source: Open Life Sci. 2024 Aug 6;19(1):20220928. doi: 10.1515/biol-2022-0928 (PMC11306960; doi:10.1515/biol-2022-0928)
Supplement: Supplementary Table [file biol-2022-0928-sm.pdf]

Supplementary material

Table S1: Primer sequences used in this study

Gene	Sequences
IL-β1-forward	5′-GAAATGCCACCTTTTGACAGTG-3′
IL-β1-reverse	5′-TGGATGCTCTCATCAGGACAG-3′
TNF-α-forward	5′-CCTCTCTCTAATCAGCCCTCTG-3′
TNF-α-reverse	5′-GAGGACCTGGGAGTAGATGAG-3′
MCP-1-forward	5′-CAGCCAGATGCAATCAATGCC-3′
MCP-1-reverse	5′-TGGAATCCTGAACCCACTTCT-3′
iNOS-forward	5′-CAGGGTGTTGCCCAAACTG-3′
iNOS-reverse	5′-GGCTGCGTTCTTCTTTGCT-3′
IL-6-forward	5′-ACTCACCTCTTCAGAACGAATTG-3′
IL-6-reverse	5′-CCATCTTTGGAAGGTTCAGGTTG-3′
CD206-forward	5′-TCCGGGTGCTGTTCTCCTA-3′
CD206-reverse	5′-CCAGTCTGTTTTTGATGGCACT-3′
Arginase1-forward	5′-GTGGAAACTTGCATGGACAAC-3′
Arginase1-reverse	5′-AATCCTGGCACATCGGGAATC-3′
IL-10-forward	5′-TACCACCTCCCGAAAATGTCA-3′
IL-10-reverse	5′-CCCAGTCTGAATGCTCATCTG-3′
ACLY-forward	5′-TCGGCCAAGGCAATTTCAGAG-3′
ACLY-reverse	5′-CGAGCATACTTGAACCGATTCT-3′
ACC1-forward	5′-CATTTTCGGTCAGGAAGAATTGC-3′
ACC1-reverse	5′-TGGAAGCATTATTACCACGAAGG-3′
GPAM-forward	5′-GATGTAAGCACACAAGTGAGGA-3′
GPAM-reverse	5′-TCCGACTCATTAGGCTTTCTTTC-3′
FASN-forward	5′-AAGGACCTGTCTAGGTTTGATGC-3′
FASN-reverse	5′-TGGCTTCATAGGTGACTTCCA-3′
ACC2-forward	5′-GAGTCAACGACCCCTGTCAAA-3′
ACC2-reverse	5′-GCTATCGGCTTGTCTTCATCGAA-3′
UCP3-forward	5′-TGTTTTGCTGACCTCGTTACC-3′
UCP3-reverse	5′-GACGGAGTCATAGAGGCCGAT-3′
Cs-forward	5′-AGTCATCGGTCAGACACCCTT-3′
Cs-reverse	5′-GTGCAGCGTTATCTCCAACAG-3′
CPT1-forward	5′-GCGCCCCTTGTTGGATGAT-3′
CPT1-reverse	5′-CCACCATGACTTGAGCACCAG-3′
HGF-forward	5′-GTGTGCCACAACTCACAACTA-3′
HGF-reverse	5′-GGTCCTGGGTATTGGAGCA-3′
VEGF-forward	5′-GAGGAGCAGTTACGGTCTGTG-3′
VEGF-reverse	5′-TCCTTTCCTTAGCTGACACTTGT-3′
Col1a1-forward	5′-GAGGGCCAAGACGAAGACATC-3′
Col1a1-reverse	5′-CAGATCACGTCATCGCACAAC-3′
TNFβ-forward	5′-ATGACACCACCTGAACGTCTC-3′
TNFβ-reverse	5′-CTCTCCAGAGCAGTGAGTTCT-3′
PDGF-forward	5′-AGCACCTTCGTTCTGACCTG-3′
PDGF-reverse	5′-TATTCTCCCGTGTCTAGCCCA-3′
GAPDH-forward	5′-TGTGGGCATCAATGGATTTGG-3′
GAPDH-reverse	5′-ACACCATGTATTCCGGGTCAAT-3′
